# Pulmonary Fibrosis in Children

**DOI:** 10.3390/jcm8091312

**Published:** 2019-08-26

**Authors:** Nadia Nathan, Chiara Sileo, Guillaume Thouvenin, Laura Berdah, Céline Delestrain, Effrosyne Manali, Spyros Papiris, Pierre-Louis Léger, Hubert Ducou le Pointe, Aurore Coulomb l’Hermine, Annick Clement

**Affiliations:** 1 Pediatric Pulmonology Department, Reference Center for Rare Lung Diseases (RespiRare), Armand Trousseau Hospital, Assistance Publique Hôpitaux de Paris (AP-HP), 75012 Paris, France; 2 Inserm UMR_S933, Sorbonne Université, 75012 Paris, France; 3 Pediatric Radiology Department, Armand Trousseau Hospital, AP-HP, 75012 Paris, France; 4 2nd Pulmonary Medicine Department, General University Hospital “Attikon”, Medical School, National and Kapodistrian University of Athens, 12462 Athens, Greece; 5 Intensive Care Unit, Armand Trousseau Hospital, AP-HP, 75012 Paris, France; 6 Pathology Department, Armand Trousseau Hospital, AP-HP, 75012 Paris, France

**Keywords:** pulmonary fibrosis, interstitial lung disease, children, usual interstitial pneumonia, nonspecific interstitial pneumonia

## Abstract

Pulmonary fibrosis (PF) is a very rare condition in children, which may be observed in specific forms of interstitial lung disease. None of the clinical, radiological, or histological descriptions used for PF diagnosis in adult patients, especially in situations of idiopathic PF, can apply to pediatric situations. This observation supports the view that PF expression may differ with age and, most likely, may cover distinct entities. The present review aims at summarizing the current understanding of PF pathophysiology in children and identifying suitable diagnostic criteria.

## 1. Introduction

In children, pulmonary fibrosis (PF) is a very rare condition, which has been sparsely described in specific forms of children’s interstitial lung disease (chILD). chILD has a reported incidence of 1–4 per millions of children and covers heterogeneous disorders in the immunocompetent host, such as surfactant disorders, pulmonary alveolar proteinosis (PAP), alveolar hemorrhage, neuroendocrine cell hyperplasia of infancy (NEHI), sarcoidosis, lung involvement of connective tissue diseases, hypersensitivity pneumonitis, and more than 25% of undefined chILD. Various classifications have been proposed so far based on clinical or histological features [1,2,3,4,5,6]. However, none of them have identified PF as a distinct chILD entity, and unlike adult PF, no PF diagnosis criteria have been proposed in children.

In adults, the most frequent adult PF, but also the most severe, is idiopathic PF (IPF), diagnosed on a usual interstitial pneumonia (UIP) pattern. UIP is characterized on high resolution computed tomography (HRCT) scan by honeycombing together with traction bronchiectasis and a subpleural and lower lobe repartition of the lesions [7,8,9]. On lung tissues, UIP is characterized by dense fibrosis with architectural distortion, predominant subpleural and/or paraseptal distribution of fibrosis, hyperplasic type 2 alveolar epithelial cells (AEC2), and fibroblastic foci with extracellular matrix (ECM) deposition in the absence of features suggesting an alternative diagnosis. Nonspecific interstitial pneumonia (NSIP) can also be associated with PF. NSIP is described on lung HRCT scans as a combination of ground-glass opacities (GGOs) and reticulations with no obvious gradient. In NSIP, basal GGOs tend to predominate over reticular opacities, with traction bronchiectasis only in advanced fibrotic-type disease [8]. On lung biopsy, NSIP is characterized by the absence of parenchymal distortion, a diffuse thickening of alveolar walls, inflammatory cell recruitment, mild fibroblastic activation, mild collagen deposits, and a relative respect of the capillary bed [9,10,11].

According to pediatric publications on chILD pathophysiology, lung fibrosis is a “destruction of pulmonary architecture caused by an abnormal wound repair response that ultimately leads to scar formation, organ malfunction, disruption of gas exchange, and respiratory failure” [12]. This definition mixes clinical, functional, and pathological issues of lung fibrosis and highlights pediatricians’ confusion over lung fibrosis’s definition. This review aims to explore the current knowledge on PF epidemiology, patterns, evolutive aspects through age, and natural history.

## 2. PF Reports in Pediatric Population

The prevalence or incidence of childhood PF seems impossible to evaluate. More than a decade ago, in the major publication on chILD histopathological classification, Deutsch et al. observed only one patient with PF out of a population of 99 pediatric patients with chILD [1]. In this 22-month-old patient with a surfactant disorder due to *SFTPC* mutation, the author described an NSIP pattern, together with Periodic acid–Schiff (PAS)-positive staining, consistent with alveolar proteinosis. The association of PF in children with surfactant disorders was also highlighted by Dishop in 2011, who suggested that PF, characterized by the presence of honeycombing, could be an end-stage complication of specific ILD conditions in older children and adolescents [13]. The lung biopsy of a teenage patient with idiopathic juvenile arthritis presenting a fibrosing NSIP pattern on the biopsy was provided as an example of PF. The author also suggested that surfactant disorders and hypersensitivity pneumonitis could be predisposing conditions for PF evolution. Later on, the rarity of PF in children was confirmed by a large study by Rice et al., who reviewed the lung biopsies of 211 patients with various forms of chILD [4]. A PF pattern was found in only 2% of the 93 patients aged under 2 years and in 7% of the 118 patients aged 2–18 years. NSIP was the most prevalent histologic pattern, but the authors highlighted that most pediatric patients harbor coexisting histologic patterns of ILD within the same sample, such as alveolar proteinosis, desquamative interstitial pneumonia (DIP), or follicular bronchiolitis.

Based on these very few pediatric studies, it seems that adult radiologic and histologic lung fibrosis patterns partially fail to precisely describe pediatric PF lesions and that UIP, the most common aspect of IPF, is exceptionally or never observed in childhood. Thus, a critical question is whether the natural history of childhood PF can evolve towards IPF.

**Figure 1:**
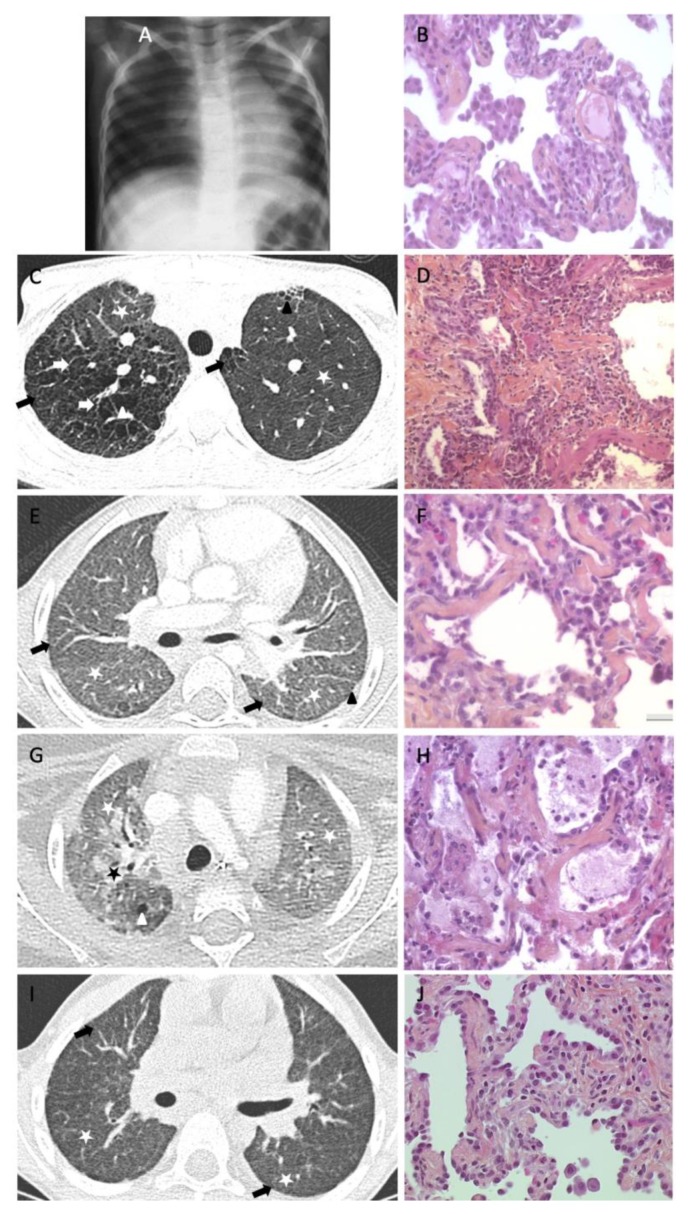
Pulmonary fibrosis examples in children. Panels A and B: Patient 1. (**A**) Chest X-ray: diffuse repartition of ground-glass opacities (GGOs). (**B**) Lung biopsy at age 6: no or mild parenchymal distortion, diffuse thickening of the alveolar walls, hyperplasic alveolar epithelial cells (AECs), inflammatory cell recruitment, mild fibroblasts activation, and mild collagen deposition. Panels C and D: Patient 2. (**C**) Transverse HRCT scan obtained at the level of the upper lobes: diffuse repartition of mild GGOs (white stars), reticulations (with interlobular septal thickening (white arrows) and intralobular lines (black arrows)), and numerous cystic lesions (white arrowheads) with focal left subpleural honeycombing (black arrowhead). Reticulations and cystic lesions are wider on the right. (**D**) Lung biopsy: parenchymal distortion, inflammatory cell recruitment, lymphoid nodules, and mild collagen and severe elastic fiber deposition. Panels E and F: Patient 3. (**E**) Transverse HRCT scan obtained under the level of the carina: diffuse repartition of GGOs (white stars), moderate reticulations (with intralobular lines (black arrows)), and few subpleural cystic lesions (black arrowhead). (**F**) Lung autopsy: no parenchymal distortion, diffuse thickening of the alveolar walls, hyperplasic AECs, mild inflammatory cell recruitment, and elastic fiber deposition. Panels G and H: Patient 4. (**G**) Transverse HRCT scan obtained at the level of the upper lobes: diffuse repartition of severe GGOs (white stars), consolidations (black star), and few cystic lesions (white arrowhead). (**H**) Postmortem biopsy: no parenchymal distortion, diffuse thickening of the alveolar walls, hyperplasic AECs, mild inflammatory cell recruitment, elastic fiber deposition, and moderate alveolar proteinosis: intra-alveolar deposit with giant cells and liproproteic material. Panels I and J: Patient 5. (**I**) HRCT scan obtained at the level of the lung bases: diffuse and homogeneous repartition of GGOs (white stars) with reticulations (with intralobular lines (black arrows)). (**J**) Lung biopsy: no parenchymal distortion, diffuse thickening of the alveolar walls, hyperplasic AECs, and moderate inflammatory cell recruitment.

As a reference center for rare lung diseases, we locally reviewed the cases of chILD who benefited from a lung sample through biopsy or autopsy. Among 119 patients, 44 underwent a lung biopsy or autopsy and only 10 were suspected of PF (Table 1). After review, only five (3%) patients were considered to meet PF criteria (i.e., fibroblast recruitment with ECM deposition). Their clinical data, HRCT scans, and lung tissue histologic analyses are provided in Table 2 and Figure 1.

## 3. Lessons from Surfactant Disorders

In the past years, for adult cases of surfactant disorders, mainly surfactant protein (SP)-C, ATP-binding cassette subfamily A member 3 (ABCA3), and NK2 homeobox 1 (NKX2-1), related diseases have been reported in sporadic or family forms of ILD. These conditions, known to be more prevalent in children than adults, are very interesting study models of lung fibrosis progression through age. The literature provides various examples of CT scan and lung tissue analyses from pediatric patients with such disorders, but very few long-term follow-ups of these patients have been reported. SP-A-related disorders have been mainly described in adults, with one pediatric case, however, in a large kindred [17,18].

In infants and childhood patients with surfactant disorders, lung imaging is heterogeneous, but diffuse GGOs and reticulations are a constant feature in severe cases. Cystic lesions, mostly with subpleural repartition, seem to appear secondarily, most likely as the consequence of a parenchymal loss of elasticity and compliance [19,20,21]. The histologic pattern of surfactant disorders is usually characteristic, with a forefront thickening of the alveolar walls; hyperplasic AEC2 bulging into the alveolar space; intramacrophagic alveolar proteinosis filling the alveolar space, sometimes associated with cholesterol clefts; and inflammatory cell recruitment. Collagen and ECM deposition are absent or moderate, as well as fibroblastic recruitment. There is usually no parenchymal destruction. Altogether, this histologic pattern is, in most cases, mimicking NSIP with additional features of alveolar proteinosis [22]. In the collaborative European network for chILD, among 24 patients with homozygous or compound heterozygous *ABCA3* mutations retrieved from the Kids Lung Register experience, NSIP, DIP, and chronic pneumonitis in infancy were the most common histologic patterns, and only one case of interstitial fibrosis pattern was reported in a patient whose lung disease was fatal in infancy [23].

In adult patients, SP-C, ABCA3, and SP-A disorders have been reported [24]. In SP-C and ABCA3 cases, lung imaging found subpleural predominance of reticulations and cystic lesions and few or no GGOs. This atypical aspect was further described as “combined pulmonary fibrosis and emphysema” (CPFE) by Cottin et al., who reported a 32-year-old patient with the most frequent p.Ile73Thr *SFTPC* mutation [25]. CPFE was further reported in adult patients with SP-C disorders, including a large kindred with patients aged 14–68 years at diagnosis, all presenting with UIP pattern [26]. Epaud et al. also reported a 41-year-old patient with CPFE related to biallelic *ABCA3* mutations with a life-long history of lung disease [27]. In these adult reports of surfactant disorders, the lung tissue analysis was characterized by parenchymal destruction, hyperplasic AEC2, but also fibroblast foci and elastic fiber deposition. Thus, both imaging and histologic pattern evocated UIP despite exceptional honeycombing. Interestingly, in large next generation sequencing (NGS) or whole exome sequencing (WES) studies including IPF patients with a histologic UIP attested by the ATS/ERS criteria (2011), a few *SFTPC* and biallelic *ABCA3* mutations were identified [11,28]. SP-A-related disorders were more recently associated with ILD [17,18,28,29]. They were alternatively associated on HRCT scans and histology of the lung with UIP, NSIP, and even DIP patterns [30]. Altogether, even if IPF terminology is evocated in adult forms of surfactant-related diseases, it seems that the histologic patterns cannot be defined only as UIP.

Little is known about surfactant disorder progression from childhood to adulthood. A few reports of family cases including children and adult presentations have been reported and even fewer descriptions of a single patient’s evolution over time. Abou Taam et al. described up to 13 years of HRCT scan follow-up in a large family with the most common *SFTPC* mutation [31]. The authors clearly showed that GGO intensity and extension were decreasing over time, whereas septal thickening, subpleural and intraparenchymal cysts, and even honeycombing were appearing and increasing with age. A comparative pediatric and adult histologic description in a single family with *SFTPC* mutation was first provided by Thomas et al. [32]. The authors showed that the infant and the adult patients harbored different patterns: cellular thickening and intra-alveolar granular material consistent with cellular NSIP in the infant tissue, compared with a UIP pattern with fibroblast foci, architectural distortion, and metaplastic epithelium in the adult biopsy. This heterogeneity was also suggested by other family studies of kindred with *SFTPC* mutations [33,34]. One family with patients presenting homozygous *ABCA3* mutations aged 16–52 years at diagnosis was also described [35]. In this family, with a late onset of the disease, all the patients showed evidence of mild fibrosis on HRCT scans with architectural distortion, intra- and interlobular thickening, apical honeycombing, and no GGOs. However, the lung tissue analysis of the youngest patient showed a relative architectural conservation and a typical childhood pattern of surfactant disorders with inflammatory cell recruitment and multinucleated cells with cholesterol clefts and mild alveolar proteinosis coexisting with fibroblast foci or smooth muscle proliferation and bronchiolar metaplasia, in favor of a mixed NSIP/DIP/UIP pattern. Finally, the 20-year evolution of a patient with *ABCA3* mutations was described, with available lung histology at age 6 and 26 years [14]. The childhood lung biopsy showed AEC2 hyperplasia, diffuse septal thickening, and few intra-alveolar macrophages with no fibrosis and a similar appearance worsened by fibrosis features in adulthood. Rare family cases of brain–lung–thyroid syndrome related to *NKX2-1* mutation have been described so far. We recently described a family with a lung involvement of the disease, the child presenting with a dense GGO pattern on HRCT scan and the mother with a fibrosing evolution at age 28 years [36]. Among a large cohort study of patients with *NKX2-1* mutations, the lung HRCT scan pattern evolution was shown in a single patient over 9 years, revealing a decrease of GGOs with no cysts or reticulations [37]. Finally, in a large family with *SFTPA1* mutations associated with PF and adenocarcinoma of the lung, the histology analysis of adult patients was described as UIP pattern, whereas the lung analysis of one infant who died at 9 months of age from lung disease showed a mixed NSIP and DIP pattern [18]. Regarding these isolated reports, it seems quite impossible to define evidence on the pattern’s evolution. However, some specificities of PF natural history could be highlighted: in childhood, GGO seems predominant, together with mixed histologic patterns, and in adulthood, septal thickening, traction cysts, and honeycombing can appear, mostly related to UIP or probable UIP patterns.

## 4. Other Situations of PF Evolution through Age

Recently, autoinflammatory disorders such as *TMEM173* mutations (STING activation) and COPA syndrome appeared as newcomers in the chILD’s field and provided other examples of ILD evolution through age. More children than adults have been described. In children, the typical presentation is an ILD with a diffuse repartition of the lesions, GGOs, reticulations, but also an early appearance of cystic lesions and traction bronchiectasis [38,39]. A family case of STING disease was provided by Picard et al., who reported CT scans and lung biopsies from the affected child and mother [16]. The pediatric HRCT scan showed more GGOs, more reticulations, and less traction bronchiectasis than the adult case. Both had cystic lesions and emphysematous lesions with upper lobe predominance. Lung tissues of both patients were available and both presented various degrees of fibrosis. The parenchymal destruction was mild in the child and major in the mother; inflammatory cell recruitment was important in the child’s parenchyma and mild in the mother’s, in whom the ECM and elastic fiber deposition was predominant. Both showed lymphoid nodules that evocated the autoinflammatory etiology of the ILD. In COPA syndrome, the lung involvement is almost a constant feature. The princeps publication reported no fibrosis in patients with ILD or in patients with alveolar hemorrhage [40]. However, more HRCT scan aspects of the patients have been described since, some with fibrosis, especially in a 12-year-old patient for whom the authors provided a 3-year-old follow-up of the HRCT scan: over time, GGOs that were initially important seemed to decrease, whereas reticulations and cystic lesions were increasing [41]. This was further confirmed by a COPA syndrome study: the authors reported the HRCT scan aspects of 11 patients, in whom parenchymal cysts of variable distribution were the most frequent feature, followed by GGOs and nodules. In only one patient were HRCT scan aspects considered to be PF. Lung tissue analysis was available for 10 patients and showed a non-UIP interstitial fibrosis in only 2 patients [42]. These reports, combined with our experience with STING and COPA syndrome, suggest that parenchymal distortion and cysts can also be present at the early stages of pediatric ILD and can evolve towards PF, mainly during the second decade (personal data).

## 5. Summary of Childhood and Adult PF Comparison

Altogether, despite the paucity of described cases, current information suggests that two distinct situations may exist: diseases with onset in infancy/childhood and adult-onset diseases. The first one presents with a cellular NSIP pattern and seems to evolve with age towards a paucicellular NSIP pattern with features of UIP or probable UIP, such as fibroblast foci or smooth muscle proliferation. On the other hand, disorders with adult or late-childhood onset seem to present with a UIP or probable UIP pattern and less frequently with fibrosing NSIP [43]. Thus, although the term PF is used in children, it seems to refer to a different pattern than in adults, with more inflammatory cell recruitment and less fibroblast recruitment and ECM deposition (Table 3). The observed pattern in children would probably not have been named “fibrosis” by adult pathologists. Thus, it seems obvious that, even when being of the same genetic origin, the pathophysiological pathways of PF in children and adults are different.

## 6. Pathophysiology

The heterogeneity of ILD phenotypes, even in the same family, has been well described in surfactant disorders. However, the reason why individuals with a pathogenic mutation are able to remain asymptomatic for decades remains unknown. The role of environmental triggers, especially viral exposure in infancy, has been suggested. One could hypothesize that the pathophysiologic fibrosing process may be different when occurring on a lung in development and growth versus on grown-up, mature, and moreover senescent lung tissue. Environmental exposures may also be different in children, in whom viral infections have been suggested as potential triggers of genetically predisposed ILD, whereas tobacco smoking and occupational exposures are the main suggested triggers in IPF [44,45,46].

At the cellular level, the pathophysiology of lung fibrosis is supposed to be initiated by repeated lung aggression that impairs the functioning of three main cellular types: AEC2, fibroblasts, and alveolar macrophages. Prolonged denudation of the basement membrane after injury contributes to altered interactions between AECs and mesenchymal cells, resulting in profound modifications of cell functions with imbalanced production of polypeptide mediators, including cytokines, growth factors, oxidants, and proteases [47,48,49]. The lung lesions induce endoplasmic reticulum (ER) stress of the AEC2 that can promote cell death or cell reprogramming. Type 1 macrophages are activated toward the alveolar space. Fibroblasts as well are activated, and they proliferate into the alveolar epithelium and differentiate into a myofibroblastic pattern. Epithelial reprogramming can lead to mesenchymal differentiation, which is called epithelial-to-mesenchymal transition. In parallel, microvascular disorders are observed with local vascular leaks and intravascular coagulation with fibrin clot deposition. Altogether, an increase of the alveolar epithelium thickness is observed, constituted by cellular and ECM deposition. Ultimately, an alveolar collapse that affects the alveolar structure is observed, with re-epithelialization phenomena [50]. This pathway, which is well described in adults, has not been evaluated in children. The few observations that have been previously described sustain that, in children, the fibrosing process could end at the cellular deposition stage without evolving, in most cases, towards ECM accumulation and alveolar collapse. This hypothesis matches with what has been observed in cell and mice models of surfactant disorders, which are associated with increased ER stress, AEC2 death, and inflammatory cell recruitment [51,52]. In autoinflammatory disorders, uncontrolled IFN-gamma production is a main feature of the pathophysiology. This could explain the accelerated parenchymal destruction process (cysts) despite minor ECM deposition [16]. The alveolar collapse and the coalescence of thickened alveolar septal walls explain the reticulation bands characteristic of ILD. However, even in these inflammatory disorders, less alveolar distortion and ECM deposition is observed in children than in adult fibrosis.

In children, PF is observed at an earlier point of the pathophysiologic process. The differences that are observed compared with adult and elderly patients could thus be related to the absence of aging phenomena. Indeed, innate and adaptative immune response and wound-healing processes become less effective with increasing age. Immunosenescence affects all cell types involved in the immunomodulation process, including epithelial cells and macrophages. With age, epithelial cells become more susceptible to cellular stress, including ER and oxidative stress, which leads to chronic inflammation, abnormal remodeling, and uncontrolled apoptotic pathways [53]. Immunosenescence also induces profound modifications in fibroblast function [54]. This is well illustrated by skin wound healing in the fetus, which is characterized by complete regeneration of the skin and the absence of any scar formation. This capacity for scarless repair is lost with age and the regeneration of the tissue architecture is achieved through the formation of a scar that exceeds the wound bed. The slowing of wound healing with age may be related to changes in the activity of various regulatory factors such as fibroblast growth factor, vascular growth factor, epithelial growth factor, and transforming growth factor (TGF)-beta production [55]. Other potential mechanisms of lung reconstruction following injury during childhood include stem cells. Stem cells are the self-renewing, primitive, undifferentiated, and multipotent source of multiple cell lineages [56,57,58,59]. They are critical during development, and their lung pool, as in other organs, decrease with age, explaining the limited tissue regeneration that occurs in adults [60,61]. In addition, bone-marrow-derived stem cells (BMSCs) are known to be able to engraft in various organs, including the lung, and to differentiate into epithelial cells [62]. The ability of immortality of embryonic stem cells disappears with age, and they also show a gradual decrease of telomere length [63,64]. Shorter telomeres are found in individuals with lung disease, and telomerase mutations are reported to be associated with familial IPF. With increasing age, the limited life span of cells may thus result from a limited cell replicative ability in response to various stresses, including DNA damage, oxidants, and telomere erosion [65,66]. Senescent mechanisms are likely to be of minor impact in infant and childhood lungs, but the difficulty of lung biopsy explains why no study is available.

## 7. Treatments in Pediatric PF

Defining children’s PF characteristics is of major importance to develop targeted therapies. It seems that pediatric ILD is more responsive to therapeutic strategies than adult ILD [67,68]. However, due to the rarity of the disease, treatment options are based on limited experiences from small series, cases reports, and local habits of the reference centers without any appropriately designed clinical trial. Moreover, it is likely that on a growing lung, treatments that are used can interfere with the fibrosing evolution of the lung. Corticosteroids are currently the preferred choice for its anti-inflammatory effects, with an initial daily oral dose of 1–2 mg/kg/day of prednisolone and/or a pulsed intravenous monthly dose of 10–30 mg/kg/day for 3 days consecutively of methylprednisolone [69]. Corticosteroids are highly effective when the cellular quota is predominant and less so when ECM deposition is predominant. Corticosteroids also display effects on lung maturation. This property is well known in preterm infants, and antenatal corticosteroids are widely used to enhance lung maturation and surfactant synthesis by AEC2 in mothers at risk of premature delivery to reduce the risk of respiratory distress syndrome [70]. It has been associated with beneficial effects in almost all chILD forms, including surfactant disorders [3,21,69,71,72], hemosiderosis [73,74], but also in severe forms of neuroendocrine cell hyperplasia of infancy (NEHI) [75].

An alternative to steroids is hydroxychloroquine (HCQ), which has a recommended dose of 6–10 mg/kg/day orally [76,77,78]. The decision as to which agent to use may be guided by the local habits of the clinicians or the lung biopsy findings, with a preference for steroids in case of a large amount of cellular desquamation and inflammation and for HCQ if increased amounts of collagen representing prefibrotic change are found [76]. In case of severe disease, steroids and HCQ may be combined. Besides its effect against malaria, HCQ displays various mechanisms of immune action, such as interference with inhibition of lysosomal proteolysis, chemotaxis, phagocytosis, antigen presentation, decreasing of various cytokines, increase of interleukins, and matrix metalloproteinase inhibition [76]. In surfactant disorders, it has been shown that HCQ may inhibit the intracellular processing of the precursor protein of SP-C [79]. HCQ accumulates in blood cells and cells of various organs including the lungs, explaining why despite low plasma levels, tissue levels can be more than 10 times higher. It has been suggested that this property could enhance the cellular effect of other medications, such as corticosteroids, without increasing their systemic side effects.

In situations of the inefficiency of steroids and HCQ, but also as a first intention treatment case of autoinflammatory disorders, other drugs such as immunosuppressive, immunomodulatory, or cytotoxic agents, such as azathioprine, cyclophosphamide, cyclosporine, or methotrexate, may be used [16,38,39,42].

Other therapeutic options include macrolides. Indeed, these antibiotics have been shown to display a number of anti-inflammatory and immunomodulatory actions. Of interest is the ability of macrolides to accumulate in parenchymal cells, including epithelial cells and phagocytes [20,80].

In the future, antifibrotic therapies that are used in adult cases of IPF will certainly be proposed for children ILD with fibrosing features. They include pirfenidone, a compound with anti-inflammatory and antifibrotic properties, and nintedanib, a tyrosine kinase inhibitor initially developed as an antitumor agent, with further activity against fibroblasts through inhibition of several growth factors [81,82]. The use of such therapies in children will require better defining the criteria of PF in the pediatric population. However, despite eventual long-term side effects, it could be anticipated that targeting multiple pathophysiologic pathways could be of benefit to avoid the fibrosing evolution of ILD in children and adults [50,83,84].

## 8. Outcome of PF in Childhood

Limited information is available in the literature about the evolution of pediatric patients with PF. This is partly due to the fact that the PF definition is not consensual and these patients may represent a very small proportion of the chILD population. chILD evolution is very heterogeneous, and despite being incurable in some cases, long-term survival has been described. Our experience of histologically proven pediatric PF seems to be in favor of a severe evolution towards terminal respiratory failure in childhood (lung transplantation or death). However, when comparing patients 3–5 (Table 2), who had very similar clinical, radiological, and histologic presentations, we observed a radically different evolution of patient 5, who recovered clinically in a few years, with no clue for explaining such a disparity. Thus, despite a global poor prognosis, further studies of long-term follow-up of pediatric patients with PF are needed.

## 9. Conclusions

**Figure 2:**
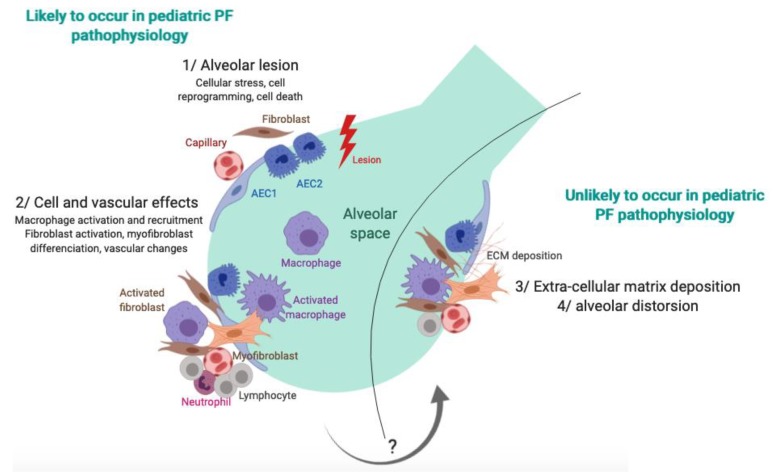
Potential pathophysiology pathway of pulmonary fibrosis in children. Repeated alveolar lesions’ effects on AECs and alveolar macrophages are likely to be observed in children and adult pulmonary fibrosis, leading to a cellular NSIP pattern. The chance of an evolution toward an adult PF pattern with more fibroblastic activation and extracellular matrix deposition remains unknown.

PF in children is poorly described and refers to a small proportion of chILD. The aggregation of national cohorts into international networks will allow pediatricians, radiologists, and pathologists to get to a consensual definition of PF criteria in children. This preliminary evaluation of available information on pediatric PF highlights that the term “pulmonary fibrosis” may have a different meaning in children than in adults, with more cellular recruitment, less collagen deposition, and less parenchymal destruction (Figure 2). Thus, unlike in adults, the hypothesis that PF is a scar state with few fibrosis lesions in children allows for considering the significant beneficial effects of antifibrotic therapies.

## Figures and Tables

**Table 1:** Suspected cases of pulmonary fibrosis (PF) in the children’s interstitial lung disease (chILD) cohort of Armand Trousseau Hospital.

chILD Condition	Number of Patients	Number of Patients with Available Lung Samples	Number of Cases with Suspected PF
Surfactant disorders	17	5	2
Autoinflammatory and systemic disorders	6	6	1
Developmental disorders	8	8	0
Others	88	25	7
**Total**	**119**	**44**	**10**

**Table 2:** Clinical data and outcomes of five patients of the Trousseau Hospital chILD cohort with lung fibrosis.

Patient Number	Clinical Presentation	Treatment	Outcome
1 [14,15]	6-year-old girl, *ABCA3*-related disease	HCQ, azithromycin	Diffuse fibrosing ILD at age 26
2 [16]	8-year-old boy, TMEM173-related disease	Corticosteroid pulses, oral corticosteroids, ruxolitinib at age 13.	Lung transplantation at age 14, died at age 16 after second lung transplantation
3	3-year-old boy, undefined chILD	Corticosteroids	Died at age 3 from respiratory failure
4	2-year-old girl, undefined chILD	Corticosteroid pulses, oral corticosteroids, azithromycin, immunosuppressive drugs	Died at age 2 from respiratory failure
5	2-year-old girl, undefined chILD	Corticosteroid pulses, oral corticosteroids, azithromycin	Asymptomatic at age 8

Abbreviations: chILD, children interstitial lung disease; HCQ, hydroxychloroquine.

**Table 3:** Childhood pathological findings of pulmonary fibrosis compared to adults.

	Pediatric PF	Adult IPF/Probable IPF
Parenchymal distortion	+	+++
Cellular recruitment	+++	+
Extracellular matrix deposition	+	+++
Fibroblast foci	+/−	+++
Honeycombing	+/−	+++
Global pattern	Predominant NSIP mixed with alveolar proteinosis, DIP, and follicular bronchiolitis	Predominant UIP pattern

+/−: absent or moderate, +: moderate, +++: important; Abbreviations: PF, pulmonary fibrosis; IPF, idiopathic pulmonary fibrosis; NSIP, nonspecific interstitial pneumonia; DIP, desquamative interstitial pneumonia; UIP, usual interstitial pneumonia.
